# Preeclampsia and academic performance in children: A nationwide study from Iceland

**DOI:** 10.1371/journal.pone.0207884

**Published:** 2018-11-21

**Authors:** Fridgeir A. Sverrisson, Brian T. Bateman, Thor Aspelund, Sigurgrimur Skulason, Helga Zoega

**Affiliations:** 1 Centre of Public Health Sciences, Faculty of Medicine, University of Iceland, Reykjavik, Iceland; 2 Division of Pharmacoepidemiology and Pharmacoeconomics, Brigham and Women’s Hospital, Department of Medicine, Harvard Medical School, Boston, Massachusetts, United States of America; 3 Department of Anesthesiology, Perioperative, and Pain Medicine, Brigham and Women’s Hospital and Harvard Medical School, Boston, Massachusetts, United States of America; 4 Icelandic Heart Association Research Institute, Kopavogur, Iceland; 5 Directorate of Education, Kopavogur, Iceland; 6 Faculty of Psychology, University of Iceland, Reykjavik Iceland; 7 Medicines Policy Research Unit, Centre for Big Data Research in Health, University of New South Wales, Sydney, Australia; University of Haifa, ISRAEL

## Abstract

**Background:**

Hypertensive disorders complicate up to 10% of pregnancies. Evidence suggests a potential association between maternal hypertensive disorders during pregnancy, particularly preeclampsia, and adverse neurodevelopment in the offspring, but existing studies are subject to limitations. We aimed to assess whether *in-utero* exposure to preeclampsia/eclampsia negatively impacts academic performance at ages 9, 12 and 15 years.

**Methods:**

Using individually linked, nationwide data from the Icelandic registries we followed all children born in 1989–2004 (N = 68,580), from birth until the end of 2014, thereof 63,014 (91.9%) took at least one standardized test. Using a stepwise, mixed-effects approach, we modelled the hypothesized relationship while adjusting for maternal, perinatal and childhood variables of interest. We compared test scores, measured on a normalized scale ranging from 0–60 with a mean of 30 and a standard deviation of 10, in the 4^th^, 7^th^, and 10^th^ grades, between children exposed to preeclampsia or eclampsia *in-utero* versus children from normotensive pregnancies in the population.

**Results:**

Children exposed to preeclampsia/eclampsia scored lower than those unexposed in mathematics across all grade levels, corresponding to a difference of 0.44 points (95% CI: 0.00, 0.89), 0.59 points (95% CI: 0.13, 1.06) and 0.59 points (95% CI: 0.08, 1.10), respectively. No differences were observed in the language arts.

**Conclusions:**

Our findings suggest a minimal effect of maternal preeclampsia/eclampsia on children’s academic performance at ages 9, 12 and 15 years. The differences observed in mathematic scores between exposed and unexposed children were minimal, less than one tenth of a standard deviation per measurement occasion.

## Introduction

Hypertensive disorders of pregnancy, which complicate up to 10% of pregnancies [1, 2], account for a substantial proportion of maternal and neonatal morbidity and mortality worldwide[3–7]. Maternal hypertension during pregnancy can be due to either pregnancy specific etiologies including gestational hypertension and preeclampsia/eclampsia or pre-existing chronic hypertension. Some, but not all [8], studies have suggested that hypertensive disorders during pregnancy may adversely affect neurodevelopment during childhood and into early adulthood, including IQ scores[9–13] and verbal ability [14, 15]. The etiology behind this relationship is largely unclear, but preeclampsia has been associated with an adverse intrauterine environment characterized by ischemia and reduced placental blood flow which may, in turn, lead to lower neurodevelopmental functioning [16].

The association between hypertensive disorders of pregnancy and impaired neurodevelopment in the offspring remains controversial. In general, studies with small sample sizes have not supported such an association [8, 17], while studies with longer-term follow-up and adequate statistical power to detect small effects, have demonstrated significant associations [9, 14, 15]. Tuovinen et al.[18] argued in their systematic review that small sample sizes, lack of confounder control and heterogeneity of research methods may explain some of the discrepancies in previous findings on this topic. Additionally, in earlier research neurocognitive outcomes have primarily been measured at a single point in time ranging, i.e. once during early childhood, adolescence or early adulthood, rather than on multiple occasions across time.

Leveraging the homogenous setting and national registration of health and academic outcomes in Iceland, we aimed to prospectively assess whether *in-utero* exposure to preeclampsia or eclampsia affected cognitive outcomes in children, measured as performance on standardized tests in the language arts and mathematics at ages 9, 12 and 15 years. Considering potential confounders and mediators, we hypothesized that children exposed to preeclampsia or eclampsia were more likely to score lower on these tests, compared with children in the general population born after a normotensive pregnancy.

## Methods

### Design, setting and population

We conducted a nationwide register-based study including all live-born infants in Iceland between January 1^st^ 1989 and December 31^st^ 2004, followed from birth until December 31^st^ 2014. We linked data from three Icelandic nationwide registers via maternal and infant personal identification numbers, uniquely assigned to each resident at birth or immigration to the country: 1) The Medical Birth Register, which has complete coverage of all live- and stillbirths in Iceland weighing over 500g or having gestational age ≥22 weeks. 2) The Directorate of Education which handles the National Examinations, a nationwide standardized testing battery mandatory for all children in elementary schools. 3) The National Medicines Registry, which contains individual level data on drugs dispensed to the total outpatient population in Iceland.

Each individual child had a maximum follow-up time of 17 years, and a minimum of 8 years. Our initial population consisted of 68,580 children; excluding those who were only present in either the Medicinal Birth Register or data on the National Examinations, yielded a population of 63,014 children available for the main analysis. Children (n = 5,566) with missing data on all outcome measurement occasions (i.e. only present in the Medical Birth Register), were included in a preliminary analysis to assess potential differences in exposure status of children with and without available data on academic outcomes.

### Definition of outcomes

The primary study outcomes were results from language arts and mathematics examinations, mandatory for all children in Iceland in 4^th^, 7^th^ and 10^th^ grade, generally corresponding to ages 9, 12 and 15, respectively.

Children in Iceland are required by law to attend school from age 6 to 16 where they progress through 10 grades. All schools at this compulsory level adhere to a national school curriculum based on laws and regulations set by the Ministry of Education, Science and Culture.

Academic performance is assessed at a national level with standardized tests in language arts and mathematics in the 4^th^, 7^th^ and 10^th^ grade. The Directorate of Education, administrates the tests, which are mandatory and held once a year at the beginning of the school year. Participation is high (over 90%); non-participation or missing test scores relate to children’s disability, illness or unspecified absence on testing day, emigration from Iceland and death.

The tests are divided into sub-domains within each subject, as demonstrated in the Supporting Information (S1 Table). Test scores are presented on a standardized normally distributed scale ranging from 0–60 with a mean of 30 and a standard deviation of 10; this scaling is achieved by combining score points or stretching them apart until the shape of the test score distribution approaches the normal distribution. This normalized scaling minimizes the effect of varying test difficulty between calendar years, making the results comparable from one year to the next.

### Definition of exposure

Data on maternal hypertensive disorders during pregnancy were retrieved from the Medical Birth Register, in which medical conditions occurring during pregnancy and childbirth are recorded according to the International Classification of Disease, currently in its 10^th^ revision [ICD-10] [19]. Conditions recorded by earlier revisions had been converted to ICD-10 codes directly into the register. In this study a child was considered exposed if the mother had a recorded diagnosis of preeclampsia, preeclampsia superimposed on chronic hypertension or eclampsia after the 20^th^ week of gestation of the respective pregnancy. We merged the diagnostic codes [ICD-10 codes O14, O11, O15] for these three conditions, into a single variable for analytical purposes, hereafter referred to as preeclampsia.

Due to changes in diagnostic- and registration criteria for chronic- and gestational hypertension occurring during the study period (e.g. new ICD codes in 1997), we excluded children born to mothers with these diagnoses [ICD-code O10, O12, O13 or O16] from all analyses (n = 1,273). A child was therefore considered as unexposed if the mother had no registered diagnosis of the abovementioned hypertensive disorders during pregnancy (normotensive pregnancy).

We previously assessed the validity of the registration of hypertensive disorders in the Medical Birth Register by comparison of recorded blood pressure values in maternity charts at women’s first and last prenatal visits [20]. In general, the quality of the Nordic Medical Birth Registers is high [21], with high positive predictive values (82% [22], 84% [23], 88% [24]) and specificity (99% [23]) for preeclampsia/eclampsia recording but lower sensitivity (43% [23], 54% [24]).

### Covariates

From the Medical Birth Register we obtained information on maternal age at delivery (in years), maternal marital status (married/cohabiting; not married; widowed/divorced), maternal occupational status (employed; student; unemployed/welfare, other aid; homemaker), maternal citizenship (Icelandic; foreign), parity (primipara; multipara), gestational age at birth (in days), singleton birth (yes; no), place of birth (urban; rural), year of birth (in years), infant sex (boy; girl), small for gestational age ([SGA] no; yes) and 5-minute Apgar score (≥7; <7). Gestational age was determined by ultrasound measurements made during or before the 20^th^ week of gestation. SGA was defined as birthweight more than 2 standard deviations below the population average based on Marsal et al.[25] All variables extracted from the Medical Birth Register were recorded during pregnancy or childbirth.

We also took attention-deficit/hyperactivity disorder (ADHD) into account, as hypertensive disorders in pregnancy have been associated with an increased risk ADHD in the offspring [26, 27] and ADHD may negatively affect children’s academic performance [28]. To identify children with a probable ADHD diagnosis, we used filled prescription for ADHD medication (code N06BA of the Anatomical Therapeutic Chemical classification system [29]). In Iceland, a prerequisite for receiving such medication is a verified ADHD diagnosis made by a pediatric, psychiatric, or neurologic specialist [30].

We obtained information on year and place of test administration (urban; rural) and when the child took the test relative to peers of the same age (concurrent with peers; ahead of peers; behind peers) from the Directorate of Education. These were recorded at each measurement occasion (at age 9, 12 and 15 years) and considered as time-varying covariates in the analysis.

### Analysis

We merged the data from the Directorate of Education with the data from the Medical Birth Register. After tidying our data by removing duplicated exam entries and excluding children only present in one of the two data sources, either missing from the dataset of academic scores or the Medical Birth Register we had a dataset of children that had at least one outcome measurement on either of the outcomes, mathematics or the language arts.

We first described the distribution of exposed and unexposed children by perinatal and demographic characteristics. We then compared the crude mean scores in mathematics and language arts at age 9, 12 and 15 years according to children’s exposure status.

For the main study analysis, we utilized linear mixed effects models to model differences in mean test scores between exposed and unexposed children across grade levels while adjusting for covariates. In the analyses, we considered gestational age, and 5-minute Apgar scores [31] as possible mediators in the hypothesized association, as they could be part of the causal mechanism between exposure to preeclampsia, characterized by inhospitable intra-uterine environment, and subsequent neurodevelopment. Similarly, ADHD was also considered as a potential mediator of the association of interest [26–28]. These variables were then introduced separately. The adjusted model yielded parameter estimates for the fixed effects of the exposure on test outcomes at each grade level and an interaction term between exposure and grade level, while adjusting for all relevant covariates. The model included a random effect associated with each child. This approach is robust to the unbalanced nature of our data where not all participants share the same outcome measurement pattern due to attrition [32].

For our main analysis we utilized a top-down modelling strategy, identical for the language arts and mathematics. Following the procedures of Singer and Willett [33] we fitted an unconditional means model, to assess intra- and inter-individual outcome variation, before introducing exposure status and grade and level to assess the crude association between exposure and mean test scores.

All available (time invariant and time varying) covariates, apart from suspected mediators, were then introduced simultaneously, including birth year and when the child took the test relative to peers of the same age, into our models to control for potential cohort effects and to estimate the relationship between exposure and outcome independently, before introducing place of test administration as a measurement level covariate. Next, we dropped non-significant fixed-effect parameters, first by verifying interactions with grade level before testing associations with mean test scores. As a last step in model definition, we introduced the suspected mediators to assess the direct effect of exposure on academic outcomes. We used deviance information criteria and Akaike’s information criteria to compare model fit through our modelling process using maximum likelihood estimation methods.

For graphical analysis all covariates are centered on their reference categories in cases of categorical predictors and mean values in cases of numerical predictors, except for birth year of child which is centered on the minimum value. The reference categories can be observed as the first listed category in Table 1.

**Table 1:** Maternal, perinatal and child characteristics by exposure status.

	Normotensive (N = 60988)	Preeclampsia/eclampsia (N = 2026)
**Continuous variables**	M (SD)	M (SD)
Maternal age at birth (years)	28.3 (5.6)	27.4 (5.9)
Gestational age at birth (days)	279.3 (11.6)	268.2 (19.8)
**Categorical variables**	*n* (%)	*n* (%)
Maternal marital status ^a^		
Married/cohabiting	55798 (91.5)	1822 (89.9)
Not married	4262 (7.0)	182 (9.0)
Widowed/divorced	917 (1.5)	22 (1.1)
Maternal occupational status ^a^		
Employed	43093 (71.7)	1464 (73.2)
Student	6938 (11.5)	322 (16.1)
Unemployed/welfare, other aid	754 (1.3)	37 (1.8)
Homemaker	9329 (15.5)	178 (8.9)
Maternal citizenship ^a^		
Icelandic	59832 (98.1)	1985 (98.0)
Foreign	1152 (1.9)	41 (2.0)
Place of birth		
Urban	41280 (67.7)	1680 (82.9)
Rural	19698 (32.3)	346 (17.1)
Parity		
Primipara	22136 (36.3)	1209 (59.7)
Multipara	38852 (63.7)	817 (40.3)
Singleton birth		
Yes	59109 (96.9)	1831 (90.4)
No	1879 (3.1)	195 (9.6)
Infant sex		
Boy	30847 (50.6)	1028 (50.7)
Girl	30141 (49.4)	998 (49.3)
Small for gestational age		
No	60058 (98.5)	1827 (90.2)
Yes	930 (1.5)	199 (9.8)
5-minute Apgar score		
≥7	59783 (98.0)	1932 (95.4)
<7	1205 (2.0)	94 (4.6)
ADHD prescriptions		
Not present	54901 (90.0)	1807 (89.2)
Present	6087 (10.0)	219 (10.8)
Place of test administration ^b^		
Urban	36253 (59.6)	1255 (62.0)
Rural	24618 (40.4)	769 (38.0)
Test participation relative to peers		
Concurrent with peers	59129 (97.0)	1969 (97.2)
Ahead of peers	1783 (2.9)	52 (2.6)
Behind peers	76 (0.1)	5 (0.2)

SD, standard deviation; ADHD, attention-deficit/hyperactive disorder

^a^ Some values were missing from the Medical Birth Register on maternal marital status (exposed n = 11), maternal occupational status (exposed n = 874; unexposed n = 25) and maternal citizenship (exposed n = 4).

^b^ Some values were missing from the Directorate of Education on place of test administration (exposed n = 117; unexposed n = 2).

We used R version 3.2.5 [34] and lme4 [35] for our main analysis and ggplot2 [36] for graphical representations.

This study was approved by the Icelandic National Bioethics Committee (VSNb2012040011/03.07) and Data Protection Authority (2014081095TS/—). The parliaments in the Nordic countries have on behalf of their populations given informed consent to be included in the national registers and the information recorded can be used for research purposes. Therefore, we did not obtain an informed written consent from participants in the study population. All personal information was anonymized and de-identified prior to analysis.

## Results

### Cohort characteristics

Of the 68,580children in our initial study population, 5,566 (8.1%) had no available academic outcome measures and were therefore not part of the main analysis. The prevalence of exposure to preeclampsia did not vary between children with and without recorded academic outcomes (3.2% vs. 3.4%). The distribution of missing academic data is presented in the Supporting Information (S2 Table).

Among the 63,014 children available for analysis, 2,026 (3.2%) were exposed to preeclampsia *in-utero*. As demonstrated in Table 1, several maternal and perinatal characteristics varied by exposure status. Children exposed to preeclampsia had a lower maternal age and shorter gestational period compared with those unexposed. Moreover, they were more likely to be SGA, have lower Apgar scores and be born in urban places than those unexposed (Table 1). The distribution of exposure by birth year is presented in the Supporting Information (S3 Table).

### Academic outcomes

Crude comparisons of mean test scores in the language arts and mathematics among exposed versus unexposed children, revealed no differences in mean scores at age 9, 12 or 15 years (Table 2).

**Table 2:** Crude mean scores and standard deviations in the language arts and mathematics by children’s exposure status.

	Language arts		Mathematics	
	Normotensive	Preeclampsia/eclampsia	Normotensive	Preeclampsia/eclampsia
	Mean (SD)	Mean (SD)	Mean (SD)	Mean (SD)
4^th^ grade (age 9)	30.1 (9.9)	30.1 (9.9)	30.0 (9.9)	29.4 (9.9)
7^th^ grade (age 12)	30.2 (9.9)	30.1 (10.0)	30.0 (9.9)	29.3 (10.1)
10^th^ grade (age 15)	30.0 (9.8)	30.4 (10.2)	29.9 (9.9)	29.9 (9.9)

SD, standard deviation

Comparing the unconditional mean models with the added grade level and exposure models, we observed lower model information criteria values when grade level and exposure status were included, indicating a better model fit.

After centering and adjusting for all relevant covariates, apart from suspected mediators and time-varying factors, and comparing those models with models where we had included place of test administration as a time-varying predictor, we observed minor fluctuations in parameter estimates. In the language arts the effects of birth place on the outcome were nullified by the inclusion of place of test administration, the interaction between place of birth and grade level was therefore dropped from subsequent models. In mathematics the interaction between birth year and grade level was dropped, the same applied to maternal citizenship for both the language arts and mathematics.

A fully adjusted model comparing the overall differences between exposure groups, across all grade levels combined resulted in differences of 0.20 points (95% CI: -0.20, 0.60) in the language arts and 0.54 points (95% CI: 0.14, 0.95) in mathematics. Indicating that unexposed children scored slightly higher on average on both academic subjects, although the difference was only significant in mathematics (data not shown).

The final model containing adjusted parameter estimates comparing exposed and unexposed children grouped by grade level showed slight outcome differences in mathematics but not in the language arts (Table 3). In mathematics children exposed to preeclampsia scored significantly lower on average on each measurement occasion, at age 9, 12 and 15 years; the observed differences were 0.44 points (95% CI: 0.00, 0.89), 0.59 points (95% CI: 0.13, 1.06) and 0.59 points (95% CI: 0.08, 1.10), respectively.

**Table 3:** Adjusted^a^ mean scores and differences in mean scores with 95% confidence intervals in the language arts and mathematics by children’s exposure status.

	Normotensive	Preeclampsia/eclampsia		
	Estimate	Estimate	Difference	95% CI
Language arts				
4th grade (age 9)	22.9	22.7	0.23	-0.20–0.66
7^th^ grade (age 12)	22.9	22.6	0.31	-0.14–0.76
10^th^ grade (age 15)	22.0	21.9	0.06	-0.42–0.55
Mathematics				
4^th^ grade (age 9)	23.0	22.6	0.44	0.00–0.89
7^th^ grade (age 12)	21.9	21.3	0.59	0.13–1.06
10^th^ grade (age 15)	20.7	20.1	0.59	0.08–1.10

^a^ Adjusted for the effects of maternal citizenship, maternal age, maternal marital status, maternal occupation, parity, singleton birth, birth year, birth place, ADHD diagnosis, place of test administration, 5-minute Apgar score, SGA status, gestational age at birth and whether the child took the test concurrent, ahead or behind peers of the same age.

**Fig 1:**
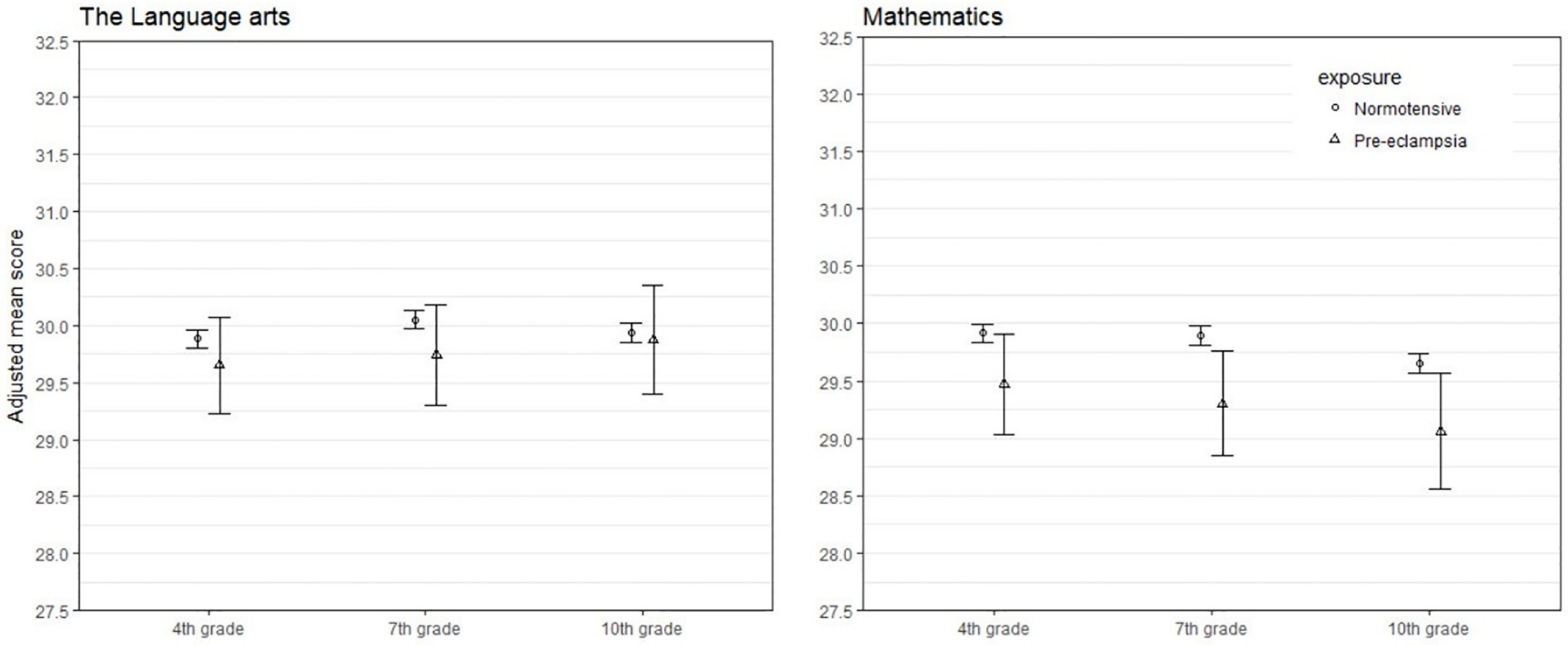
Marginal predicted scores^a^ in language arts and mathematics by exposure to preeclampsia/eclampsia.

Fig 1 shows marginal predicted values and corresponding 95% confidence intervals between academic subjects for children exposed to preeclampsia and unexposed children with all covariates centered on their reference categories in cases of categorical predictors and mean values in cases of numerical predictors, except for birth year of child which is centered on the minimum value. The reference categories can be observed as the first listed category in Table 1.

## Discussion

Our objective with this prospective population-based cohort study was to assess whether hypertensive disorders during pregnancy, specifically preeclampsia, affect academic performance in offspring as measured by standardized tests in the 4^th^, 7^th^ and 10^th^ grades of elementary schools in Iceland. Overall, our findings suggest that the effects of preeclampsia/eclampsia on academic performance are very small. Exposed children scored on average slightly, yet significantly, lower on mathematics tests in at age 9, 12 and 15 years, *vis-à-vis* their unexposed counterparts, but this difference is unlikely to have clinical implications.

Our study builds upon Barker’s landmark publication [14] from 1967 on obstetric complications and school performance, suggesting that toxaemia (preeclampsia) was associated with slightly impaired verbal reasoning at age 11, independent of gestational age at birth and birth order. Subsequent studies with short-to medium term follow-up periods have generally not detected differences in cognitive abilities by exposure to hypertensive disorders *in-utero* [9, 15, 37]. But studies extending into early [13] and late [38] adulthood have reported small differences in cognitive functioning, including in arithmetic reasoning [39]. Tuovinen et al. [18] speculated in their systematic review of maternal hypertensive pregnancy disorders and cognitive functioning in the offspring whether this is due to the effects of maternal hypertension manifesting later in life or merely due to a cohort effect reflected in improved treatment options. Due to the longitudinal and progression focused nature of our analysis, our findings are informative on this matter as they indicate that the effects of maternal preeclampsia on outcomes in mathematics are present at age 9, 12 and 15 years. But it is unclear why mathematics but not language abilities would be affected by an inhospitable intra-uterine environment.

This study has three prominent strengths. First, the large population-based study sample gave us ample precision to detect any hypothesized effects of preeclampsia/eclampsia on academic outcomes. Given the nature of the study we believe the findings to generalizable to populations beyond Iceland. Second, the longitudinal nature of our study is especially appropriate for documenting progress of this kind and in covering the wide range of individual characteristics both time-invariant and time-variant making adjustment for these factors possible. Third, the outcome measurements are well suited for the study analysis, comprising individual rankings on standardized test in two core subjects, measured on three occasions for the total study population.

The major limitations of the present study include a lack of information on maternal BMI, smoking and co-morbidity, such as diabetes—a known risk factor for preeclampsia [40]. Evidence suggests that smoking during pregnancy may be inversely associated with preeclampsia [41], and obesity is a strong risk factor for hypertension [20, 42]. Maternal BMI has also been linked to child cognitive development including emotional symptoms, conduct problems and autism [43], while maternal obesity has been associated with lower overall IQ scores (3.2 points) in children [44]. Similar associations have also been observed with maternal smoking [45]. Accounting for maternal lifestyle and morbidity of this sort, would have been optimal to avert residual confounding and to improve our understanding of the association under study. Further, our analysis was based on children who participated in at least one standardized test. Despite the high participation rate (>90%), non-participation due to children’s disability, death or emigration may have biased our results towards the null. Finally, misclassification of children’s exposure status is possible. We attempted to circumvent this by focusing on diagnoses with high specificity and excluded from the analysis children with maternal records of other hypertensive disorders (i.e. gestational hypertension or chronic hypertension, essential and secondary). Nevertheless, having accurate information on all hypertensive disorders in pregnancy would have yielded a more complete picture of the hypothesized association.

### Conclusion

Overall our findings suggest a minimal, or no, effect of maternal preeclampsia/eclampsia on children’s academic performance at ages 9 to 15 years. The findings are important given the high number of women dealing with hypertensive disorders in pregnancy. They also provide reassuring information to clinicians and parents about the implications of preeclampsia on the child’s development in the longer-term. In our analysis we were able to follow a nationwide cohort of children from prenatal life and measure academic performance on three separate occasions in childhood. The differences observed in mathematic scores between children exposed and unexposed to preeclampsia/eclampsia were less than one tenth of a standard deviation per measurement occasion.

## Supporting information

S1 Table The subdivisions of nationally administered standardized tests in the language arts and mathematics. (DOCX)

S2 Table Children with and without academic outcomes by exposure status. (DOCX)

S3 Table Children’s birth year by exposure status. (DOCX)

S4 Table Study variables. ICD, International Classification of Disease; SD, standard deviation; ADHD, attention-deficit/hyperactive disorder; ATC, Anatomical Therapeutic Chemical classification.
(DOCX)

## References

[1] Roberts CL, Ford JB, Algert CS, Antonsen S, Chalmers J, Cnattingius S, et al Population-based trends in pregnancy hypertension and pre-eclampsia: an international comparative study. BMJ open. 2011;1(1):e000101 Epub 2011/10/25. 10.1136/bmjopen-2011-000101 . 22021762 10.1136/bmjopen-2011-000101 PMC3191437

[2] Eiriksdottir VH, Valdimarsdottir UA, Asgeirsdottir TL, Hauksdottir A, Lund SH, Bjarnadottir RI, et al Pregnancy-Induced Hypertensive Disorders before and after a National Economic Collapse: A Population Based Cohort Study. PLoS One. 2015;10(9):e0138534 10.1371/journal.pone.0138534 . 26379126 10.1371/journal.pone.0138534 PMC4575018

[3] Duley L. The Global Impact of Pre-eclampsia and Eclampsia. Seminars in Perinatology. 2009;33(3):130–7. 10.1053/j.semperi.2009.02.010. 19464502

[4] Khan KS, Wojdyla D, Say L, Gulmezoglu AM, Van Look PF. WHO analysis of causes of maternal death: a systematic review. Lancet. 2006;367(9516):1066–74. Epub 2006/04/04. 10.1016/S0140-6736(06)68397-9 . 16581405 10.1016/S0140-6736(06)68397-9

[5] Lambert G, Brichant JF, Hartstein G, Bonhomme V, Dewandre PY. Preeclampsia: an update. Acta anaesthesiologica Belgica. 2014;65(4):137–49. Epub 2015/01/28. . 25622379

[6] Kuklina EV, Ayala C, Callaghan WM. Hypertensive disorders and severe obstetric morbidity in the United States. Obstet Gynecol. 2009;113(6):1299–306. Epub 2009/05/23. 10.1097/AOG.0b013e3181a45b25 . 19461426 10.1097/AOG.0b013e3181a45b25

[7] World Health Organization. WHO recommendations for prevention and treatment of pre-eclampsia and eclampsia. Geneva: World Health Organisation, 2011. 23741776

[8] Seidman DS, Laor A, Gale R, Stevenson DK, Mashiach S, Danon YL. Pre-eclampsia and offspring’s blood pressure, cognitive ability and physical development at 17-years-of-age. British journal of obstetrics and gynaecology. 1991;98(10):1009–14. Epub 1991/10/01. . 1751432 10.1111/j.1471-0528.1991.tb15339.x

[9] Heikura U, Hartikainen A-L, Nordstrom T, Pouta A, Taanila A, Jarvelin M-R. Maternal Hypertensive Disorders during Pregnancy and Mild Cognitive Limitations in the Offspring. Paediatric and perinatal epidemiology. 2013;27(2):188–98. 10.1111/ppe.12028 23374064 10.1111/ppe.12028

[10] Many A, Fattal A, Leitner Y, Kupferminc MJ, Harel S, Jaffa A. Neurodevelopmental and cognitive assessment of children born growth restricted to mothers with and without preeclampsia. Hypertension in pregnancy. 2003;22(1):25–9. Epub 2003/03/22. 10.1081/PRG-120016791 . 12648440 10.1081/PRG-120016791

[11] Morsing E, Marsal K. Pre-eclampsia- an additional risk factor for cognitive impairment at school age after intrauterine growth restriction and very preterm birth. Early human development. 2014;90(2):99–101. Epub 2014/01/07. 10.1016/j.earlhumdev.2013.12.002 . 24388669 10.1016/j.earlhumdev.2013.12.002

[12] Leversen KT, Sommerfelt K, Ronnestad A, Kaaresen PI, Farstad T, Skranes J, et al Prediction of neurodevelopmental and sensory outcome at 5 years in Norwegian children born extremely preterm. Pediatrics. 2011;127(3):e630–8. Epub 2011/02/16. 10.1542/peds.2010-1001 . 21321031 10.1542/peds.2010-1001

[13] Ehrenstein V, Rothman KJ, Pedersen L, Hatch EE, Sorensen HT. Pregnancy-associated hypertensive disorders and adult cognitive function among Danish conscripts. Am J Epidemiol. 2009;170(8):1025–31. 10.1093/aje/kwp223 . 19726495 10.1093/aje/kwp223

[14] Barker DJ, Edwards JH. Obstetric complications and school performance. Br Med J. 1967;3(5567):695–9. . 6068401 10.1136/bmj.3.5567.695 PMC1843017

[15] Whitehouse AJO, Robinson M, Newnham JP, Pennell CE. Do hypertensive diseases of pregnancy disrupt neurocognitive development in offspring? Paediatric and perinatal epidemiology. 2012;26(2):101–8. 10.1111/j.1365-3016.2011.01257.x 22324495 10.1111/j.1365-3016.2011.01257.x

[16] Ananth CV, Friedman AM. Ischemic placental disease and risks of perinatal mortality and morbidity and neurodevelopmental outcomes. Semin Perinatol. 2014;38(3):151–8. Epub 2014/05/20. 10.1053/j.semperi.2014.03.007 . 24836827 10.1053/j.semperi.2014.03.007

[17] Ounsted M, Moar VA, Cockburn J, Redman CW. Factors associated with the intellectual ability of children born to women with high risk pregnancies. British medical journal (Clinical research ed). 1984;288(6423):1038–41. Epub 1984/04/07. . 6423184 10.1136/bmj.288.6423.1038 PMC1442698

[18] Tuovinen S, Eriksson JG, Kajantie E, Raikkonen K. Maternal hypertensive pregnancy disorders and cognitive functioning of the offspring: a systematic review. Journal of the American Society of Hypertension: JASH. 2014;8(11):832–47.e1. Epub 2014/12/03. 10.1016/j.jash.2014.09.005 . 25455009 10.1016/j.jash.2014.09.005

[19] World Health Organization. ICD-10 Version:2016 2016 [cited 2016 01/03/2016]. http://apps.who.int/classifications/icd10/browse/2016/en#/O10-O16.

[20] Gudnadottir TA, Bateman BT, Hernadez-Diaz S, Luque-Fernandez MA, Valdimarsdottir U, Zoega H. Body Mass Index, Smoking and Hypertensive Disorders during Pregnancy: A Population Based Case-Control Study. PLoS One. 2016;11(3):e0152187 10.1371/journal.pone.0152187 . 27010734 10.1371/journal.pone.0152187 PMC4807030

[21] Langhoff-Roos J, Krebs L, Klungsoyr K, Bjarnadottir RI, Kallen K, Tapper AM, et al The Nordic medical birth registers—a potential goldmine for clinical research. Acta Obstet Gynecol Scand. 2014;93(2):132–7. 10.1111/aogs.12302 . 24237585 10.1111/aogs.12302

[22] Thomsen LC, Klungsoyr K, Roten LT, Tappert C, Araya E, Baerheim G, et al Validity of the diagnosis of pre-eclampsia in the Medical Birth Registry of Norway. Acta Obstet Gynecol Scand. 2013;92(8):943–50. 10.1111/aogs.12159 . 23621424 10.1111/aogs.12159

[23] Klungsoyr K, Harmon QE, Skard LB, Simonsen I, Austvoll ET, Alsaker ER, et al Validity of pre-eclampsia registration in the medical birth registry of norway for women participating in the norwegian mother and child cohort study, 1999–2010. Paediatr Perinat Epidemiol. 2014;28(5):362–71. 10.1111/ppe.12138 . 25040774 10.1111/ppe.12138 PMC4167249

[24] Kristensen J, Langhoff-Roos J, Skovgaard LT, Kristensen FB. Validation of the Danish Birth Registration. J Clin Epidemiol. 1996;49(8):893–7. . 8699210 10.1016/0895-4356(96)00018-2

[25] Marsal K, Persson PH, Larsen T, Lilja H, Selbing A, Sultan B. Intrauterine growth curves based on ultrasonically estimated foetal weights. Acta Paediatr. 1996;85(7):843–8. . 8819552 10.1111/j.1651-2227.1996.tb14164.x

[26] Bohm S, Curran EA, Kenny LC, O’Keeffe GW, Murray D, Khashan AS. The Effect of Hypertensive Disorders of Pregnancy on the Risk of ADHD in the Offspring. J Atten Disord. 2017:1087054717690230. 10.1177/1087054717690230 . 28162026 10.1177/1087054717690230

[27] Pohlabeln H, Rach S, De Henauw S, Eiben G, Gwozdz W, Hadjigeorgiou C, et al Further evidence for the role of pregnancy-induced hypertension and other early life influences in the development of ADHD: results from the IDEFICS study. Eur Child Adolesc Psychiatry. 2017;26(8):957–67. 10.1007/s00787-017-0966-2 . 28258320 10.1007/s00787-017-0966-2

[28] Zoega H, Rothman KJ, Huybrechts KF, Olafsson O, Baldursson G, Almarsdottir AB, et al A population-based study of stimulant drug treatment of ADHD and academic progress in children. Pediatrics. 2012;130(1):e53–62. 10.1542/peds.2011-3493 . 22732167 10.1542/peds.2011-3493

[29] World Health Organization. ATC/DDD Index 2016: World Health Organization; 2015 [cited 2016 24 May]. http://www.whocc.no/atc_ddd_index/.

[30] Icelandic Health Insurance. Vinnuregla fyrir útgáfu lyfjaskírteinis metýlfenídat 2011 [updated Working rule for reimbursement of methylphenidate; cited 2016 24 May]. http://www.sjukra.is/media/vinnnureglur-lyfjaskirteina/Metylfenidat_1.jan_2011.pdf.

[31] Stuart A, Otterblad Olausson P, Kallen K. Apgar scores at 5 minutes after birth in relation to school performance at 16 years of age. Obstet Gynecol. 2011;118(2 Pt 1):201–8. 10.1097/AOG.0b013e31822200eb . 21734618 10.1097/AOG.0b013e31822200eb

[32] Cnaan A, Laird NM, Slasor P. Using the general linear mixed model to analyse unbalanced repeated measures and longitudinal data. Statistics in medicine. 1997;16(20):2349–80. Epub 1997/11/14. . 9351170 10.1002/(sici)1097-0258(19971030)16:20<2349::aid-sim667>3.0.co;2-e

[33] Singer JD, Willett JB. Applied longitudinal data analysis: Modeling change and event occurrence. New York: Oxford University Press; 2003 644 p.

[34] R Core Team. R: A Language and Environment for Statistical Computing. Vienna, Austria 2016.

[35] Bates D, Mächler M, Bolker B, Walker S. Fitting Linear Mixed-Effects Models Using lme4. Journal of Statistical Software. 2015;67(1):1–48.

[36] Wickham H. ggplot2: Elegant Graphics for Data Analysis: Springer-Verlag New York; 2009.

[37] Leitner Y, Harel S, Geva R, Eshel R, Yaffo A, Many A. The neurocognitive outcome of IUGR children born to mothers with and without preeclampsia. The journal of maternal-fetal & neonatal medicine: the official journal of the European Association of Perinatal Medicine, the Federation of Asia and Oceania Perinatal Societies, the International Society of Perinatal Obstet. 2012;25(11):2206–8. Epub 2012/04/25. 10.3109/14767058.2012.684164 . 22524188 10.3109/14767058.2012.684164

[38] Tuovinen S, Eriksson JG, Kajantie E, Lahti J, Pesonen AK, Heinonen K, et al Maternal hypertensive disorders in pregnancy and self-reported cognitive impairment of the offspring 70 years later: the Helsinki Birth Cohort Study. Am J Obstet Gynecol. 2013;208(3):200 e1–9. 10.1016/j.ajog.2012.12.017 . 23246316 10.1016/j.ajog.2012.12.017

[39] Tuovinen S, Raikkonen K, Kajantie E, Henriksson M, Leskinen JT, Pesonen AK, et al Hypertensive disorders in pregnancy and cognitive decline in the offspring up to old age. Neurology. 2012;79(15):1578–82. 10.1212/WNL.0b013e31826e2606 . 23035059 10.1212/WNL.0b013e31826e2606 PMC3475625

[40] Ros HS, Cnattingius S, Lipworth L. Comparison of risk factors for preeclampsia and gestational hypertension in a population-based cohort study. Am J Epidemiol. 1998;147(11):1062–70. . 9620050 10.1093/oxfordjournals.aje.a009400

[41] Stone CD, Diallo O, Shyken J, Leet T. The combined effect of maternal smoking and obesity on the risk of preeclampsia. J Perinat Med. 2007;35(1):28–31. Epub 2007/02/23. 10.1515/JPM.2007.003 . 17313306 10.1515/JPM.2007.003

[42] Seely EW, Ecker J. Chronic hypertension in pregnancy. Circulation. 2014;129(11):1254–61. Epub 2014/03/19. 10.1161/CIRCULATIONAHA.113.003904 . 24637432 10.1161/CIRCULATIONAHA.113.003904

[43] Jo H, Schieve LA, Sharma AJ, Hinkle SN, Li R, Lind JN. Maternal prepregnancy body mass index and child psychosocial development at 6 years of age. Pediatrics. 2015;135(5):e1198–209. Epub 2015/04/29. 10.1542/peds.2014-3058 . 25917989 10.1542/peds.2014-3058 PMC4411780

[44] Pugh SJ, Richardson GA, Hutcheon JA, Himes KP, Brooks MM, Day NL, et al Maternal Obesity and Excessive Gestational Weight Gain Are Associated with Components of Child Cognition. The Journal of nutrition. 2015;145(11):2562–9. Epub 2015/10/02. 10.3945/jn.115.215525 . 26423736 10.3945/jn.115.215525 PMC4620725

[45] Anthopolos R, Edwards SE, Miranda ML. Effects of maternal prenatal smoking and birth outcomes extending into the normal range on academic performance in fourth grade in North Carolina, USA. Paediatric and perinatal epidemiology. 2013;27(6):564–74. Epub 2013/10/19. 10.1111/ppe.12081 . 24134528 10.1111/ppe.12081

