# Supplementary material for: Preeclampsia and academic performance in children: A nationwide study from Iceland
Source: PLoS One. 2018 Nov 21;13(11):e0207884. doi: 10.1371/journal.pone.0207884 (PMC6249018; doi:10.1371/journal.pone.0207884)
Supplement: S1 Table — (DOCX) [file pone.0207884.s001.docx]

**Supporting information**

**S1 Table. The subdivisions of nationally administered standardized tests in the language arts and mathematics.**

|  | Language arts | Mathematics |
| --- | --- | --- |
| 4^th^ grade  (age 9) | - spelling - reading comprehension - writing - grammar | - calculations and operations - geometry and measurement - numbers and comprehension |
| 7^th^ grade  (age 12) | - spelling - reading comprehension - writing - grammar/word usage | - calculations and operations - geometry and measurement - statistics |
| 10^th^ grade  (age 15) | - spelling (until 2009) - reading comprehension/literature - grammar/word usage - writing | - calculations and operations - ratios and percentages (statistics) - geometry and measurement - algebra and patterns |
