# Supplementary material for: Preeclampsia and academic performance in children: A nationwide study from Iceland
Source: PLoS One. 2018 Nov 21;13(11):e0207884. doi: 10.1371/journal.pone.0207884 (PMC6249018; doi:10.1371/journal.pone.0207884)
Supplement: S2 Table — (DOCX) [file pone.0207884.s002.docx]

**S2 Table. Children with and without academic outcomes by exposure status.**

|  | Normotensive  (N=66,363) | Preeclampsia/eclampsia  (N=2,217) |
| --- | --- | --- |
| **Academic data missing, n (%)** |  |  |
| No | 60,988 (91.9) | 2,026 (91.4) |
| Yes | 5,375 (8.1) | 191 (8.6) |
