# Supplementary material for: Preeclampsia and academic performance in children: A nationwide study from Iceland
Source: PLoS One. 2018 Nov 21;13(11):e0207884. doi: 10.1371/journal.pone.0207884 (PMC6249018; doi:10.1371/journal.pone.0207884)
Supplement: S3 Table — (DOCX) [file pone.0207884.s003.docx]

**S3 Table. Children’s birth year by exposure status.**

|  | Normotensive | | Preeclampsia/eclampsia | |
| --- | --- | --- | --- | --- |
|  | N | n (%) | N | n (%) |
| Birth year | 60988 |  | 2026 |  |
| 1989 |  | 4119 (6.8) |  | 91 (4.5) |
| 1990 |  | 4365 (7.2) |  | 78 (3.8) |
| 1991 |  | 4086 (6.7) |  | 107 (5.3) |
| 1992 |  | 4160 (6.8) |  | 130 (6.4) |
| 1993 |  | 4166 (6.8) |  | 122 (6.0) |
| 1994 |  | 4024 (6.6) |  | 152 (7.5) |
| 1995 |  | 3827 (6.3) |  | 168 (8.3) |
| 1996 |  | 3841 (6.3) |  | 223 (11.0) |
| 1997 |  | 3673 (6.0) |  | 107 (5.3) |
| 1998 |  | 3700 (6.1) |  | 108 (5.3) |
| 1999 |  | 3639 (6.0) |  | 94 (4.6) |
| 2000 |  | 3733 (6.1) |  | 97 (4.8) |
| 2001 |  | 3547 (5.8) |  | 104 (5.1) |
| 2002 |  | 3397 (5.6) |  | 150 (7.4) |
| 2003 |  | 3312 (5.4) |  | 149 (7.4) |
| 2004 |  | 3399 (5.6) |  | 146 (7.2) |
