# Supplementary material for: Preeclampsia and academic performance in children: A nationwide study from Iceland
Source: PLoS One. 2018 Nov 21;13(11):e0207884. doi: 10.1371/journal.pone.0207884 (PMC6249018; doi:10.1371/journal.pone.0207884)
Supplement: S4 Table — ICD, International Classification of Disease; SD, standard deviation; ADHD, attention-deficit/hyperactive disorder; ATC, Anatomical Therapeutic Chemical classification. (DOCX) [file pone.0207884.s004.docx]

**S4 Table. Study variables.**

| **Variable** | **Codes, values** | **Source** | **Details** |
| --- | --- | --- | --- |
| Preeclampsia | ICD-10 O14, O11, 015 | Medical Birth Register | Exposure  preeclampsia, preeclampsia superimposed on chronic hypertension, eclampsia |
| Gestational hypertension | ICD-10 O13 | Medical Birth Register | Excluded |
| Chronic hypertension | ICD-10 O10 | Medical Birth Register | Excluded |
| Test scores in 4^th^, 7^th^, 10^th^ grade (age 9, 12 15)   - Language arts - Mathematics | 0-60 standardized normally distributed scale (mean 30, SD 10) | Directorate of Education | Outcomes |
| Marital status | married/cohabiting, not married, widowed/divorced | Medical Birth Register | Covariate |
| Parity | primipara, multipara | Medical Birth Register | Covariate |
| Age at delivery | years | Medical Birth Register | Covariate |
| Citizenship | Icelandic, foreign | Medical Birth Register | Covariate |
| Occupational status | Employed, student, unemployed, welfare/other aid, homemaker | Medical Birth Register | Covariate |
| Singleton pregnancy | yes, no | Medical Birth Register | Covariate |
| Infant sex | boy, girl | Medical Birth Register | Covariate |
| Birth year | year of birth | Medical Birth Register | Covariate |
| Gestational age | number of days | Medical Birth Register | Covariate, based on ultrasound |
| ADHD prescription fills | ATC code N06BA | National Medicines Registry | Potential mediator, proxy for ADHD |
| 5-minute Apgar score | ≥7, <7 | Medical Birth Register | Potential mediator |
| Small for gestational age status (SGA) | yes, no | Medical Birth Register | Potential mediator, based on birthweight and gestational age |
| Birth place | capital region, other | Medical Birth Register | Covariate |
| Place of test administration | capital region, other | Directorate of Education | Covariate |
| Test participation relative to peers | on time, accelerated, delayed | Directorate of Education | Covariate |

ICD, International Classification of Disease; SD, standard deviation; ADHD, attention-deficit/hyperactive disorder; ATC, Anatomical Therapeutic Chemical classification
